# Medical Expert Knowledge Meets AI to Enhance Symptom Checker Performance for Rare Disease Identification in Fabry Disease: Mixed Methods Study

**DOI:** 10.2196/55001

**Published:** 2025-08-28

**Authors:** Anne Pankow, Nico Meißner-Bendzko, Jessica Kaufeld, Laura Fouquette, Fabienne Cotte, Stephen Gilbert, Ewelina Türk, Anibh Das, Christoph Terkamp, Gerhard-Rüdiger Burmester, Annette Doris Wagner

**Affiliations:** 1 Department of Rheumatology and Clinical Immunology, Charité-Universitätsmedizin Berlin, Berlin, Germany; 2 Department of Gastroneterology, Hepatology, Infectious Diseases and Endocrinology, Hannover Medical School, Hannover, Germany; 3 Ada Health GmbH, Berlin, Germany; 4 Department of Nephrology and Hypertension, Hannover Medical School, Carl-Neuberg-Strasse 1, Hannover, 30625, Germany, 49 511 532 3745; 5 Else Kröner Fresenius Center for Digital Health, TU Dresden University of Technology, Dresden, Germany; 6 Department of Paediatrics, Hannover Medical School, Hannover, Germany

**Keywords:** artificial intelligence, AI, symptom assessment, Pompe disease, Gaucher disease, Fabry disease, medical expert, app, rare diseases, lysosomal, clinical vignettes, clinical database, interviews, patient

## Abstract

**Background:**

Rare diseases, which affect millions of people worldwide, pose a major challenge, as it often takes years before an accurate diagnosis can be made. This delay results in substantial burdens for patients and health care systems, as misdiagnoses lead to inadequate treatment and increased costs. Artificial intelligence (AI)–powered symptom checkers (SCs) present an opportunity to flag rare diseases earlier in the diagnostic work-up. However, these tools are primarily based on published literature, which often contains incomplete data on rare diseases, resulting in compromised diagnostic accuracy. Integrating expert interview insights into SC models may enhance their performance, ensuring that rare diseases are considered sooner and diagnosed more accurately.

**Objective:**

The objectives of our study were to incorporate expert interview vignettes into AI-powered SCs, in addition to a traditional literature review, and to evaluate whether this novel approach improves diagnostic accuracy and user satisfaction for rare diseases, focusing on Fabry disease.

**Methods:**

This mixed methods prospective pilot study was conducted at Hannover Medical School, Germany. In the first phase, guided interviews were conducted with medical experts specialized in Fabry disease to create clinical vignettes that enriched the AI SC’s Fabry disease model. In the second phase, adult patients with a confirmed diagnosis of Fabry disease used both the original and optimized SC versions in a randomized order. The versions, containing either the original or the optimized Fabry disease model, were evaluated based on diagnostic accuracy and user satisfaction, which were assessed through questionnaires.

**Results:**

Three medical experts with extensive experience in lysosomal storage disorder Fabry disease contributed to the creation of 5 clinical vignettes, which were integrated into the AI-powered SC. The study compared the original and optimized SC versions in 6 patients with Fabry disease. The optimized version improved diagnostic accuracy, with Fabry disease identified as the top suggestion in 33% (2/6) of cases, compared to 17% (1/6) with the original model. Additionally, overall user satisfaction was higher for the optimized version, with participants rating it more favorably in terms of symptom coverage and completeness.

**Conclusions:**

This study demonstrates that integrating expert-derived clinical vignettes into AI-powered SCs can improve diagnostic accuracy and user satisfaction, particularly for rare diseases. The optimized SC version, which incorporated these vignettes, showed improved performance in identifying Fabry disease as a top diagnostic suggestion and received higher user satisfaction ratings compared to the original version. To fully realize the potential of this approach, it is crucial to include vignettes representing atypical presentations and to conduct larger-scale studies to validate these findings.

## Introduction

### Background

Taken as a group, rare diseases are common and affect about 350 million people worldwide [1]. It is estimated that 1 in 17 individuals will encounter a rare disease during their lifetime [2]. Diagnosing rare diseases continues to be a challenge for health care professionals and health care systems [3].

Patients with rare diseases often have to go through a long diagnostic journey, waiting an average of 6 years from the onset of symptoms to an accurate diagnosis [4]. For some rare diseases, the average time to diagnosis is even far beyond this—the median duration from the initial manifestation of Fabry disease to its diagnosis being approximately 10.3 (IQR 5.9-62.0) years. The mean duration from the onset of the disease to the initiation of enzyme replacement therapy takes even longer, approximately 21.3 years [5].

Rare diseases are often misdiagnosed at first, resulting in inadequate treatment, significant impairment of the patients’ quality of life, progression of their disease, and sometimes even irreversible complications [6]. Additional medical consultations and inappropriate therapies cause significant costs for both individuals and health care systems [7]. Insufficient knowledge about rare diseases and a lack of awareness are considered to be the main factors leading to delay in diagnosis, particularly in primary care. Due to their rarity, rare diseases are often overlooked by general practitioners (GPs) because of their limited knowledge [2,3]. Another challenge patients face is that there are only a handful of specialized experts for each rare disease [8], and these experts are not evenly distributed in the health care system, so that access is limited. Artificial intelligence (AI)–powered symptom checkers (SCs) have the potential to aid the detection of rare diseases, thereby reducing the time to diagnosis [2,6,9]. AI approaches, as are SCs, are increasingly implemented in health care settings to help alleviate the burden on the systems and to improve the quality of care [10]. The goal of SCs is to provide information to the users that enables them to identify the likely cause of their symptoms [11,12]. Additionally, many SCs offer triage recommendations based on these symptoms and guide patients on whether they should seek medical assistance and, if so, at what level—be it a hospital, general practice, or self-care at home—taking into account the urgency of the situation [12,13].

One such SC is Ada. Ada’s foundation draws on digitized medical knowledge, predictive algorithms, Bayesian inference, and validation against diverse case sets to deliver precise guidance [14]. The SC provides up to 5 disease suggestions as possible causes for the user’s symptoms, without claiming to replace physicians or to make a diagnosis. Similar to a physician’s initial patient history-taking process, the SC begins with gathering fundamental health information and then proceeds to ask follow-up questions based on the provided symptoms. Once the symptom assessment is completed, the user receives a structured summary report of the currently relevant symptoms, symptoms that have been ruled out, and those that remain uncertain. The SC suggests between 3 and 5 disease suggestions, along with the corresponding probabilities and recommended next steps for the user [15]. The SC is based on an ever-evolving medical database, continuously incorporating the latest research findings. Other commonly used SCs include Buoy, K Health, Mediktor, Symptomate, Your.MD, and WebMD [16].

When integrated into hospital websites or booking portals, SCs give users guidance on whether, when, and where to seek care within their network while also explaining the most likely causes of their symptoms [17,18]. By directing patients to appropriate care, hospital resources can be used more efficiently and allocated to those who are truly in need of medical attention. Triage accuracy varies depending on the SC, from 48.8% to 90.1%, and has shown to be comparable to those of telephone triage [12].

Another significant benefit of SCs is their ability to support the diagnostic accuracy of health care professionals, which is particularly relevant for rare diseases. SCs can flag potential rare diseases that might otherwise go unnoticed, prompting health care providers to consider diagnoses they may not have initially considered. By bringing these less common possibilities to the forefront, SCs can aid in the earlier detection of rare diseases, ultimately improving patient outcomes. This capability is especially valuable in complex cases where symptoms may be ambiguous or overlap with more common diseases, ensuring that rare diseases are not dismissed too quickly.

The impact of this capability is evident in a study involving 450 patients, where the SC (Ada) demonstrated a 10% improvement in physicians’ diagnostic accuracy [13]. Patients who received an early diagnosis experienced significantly fewer complications and had a shorter hospital stay (*P*<.001). Additionally, the same SC outperformed both rheumatologists and GPT-4 in diagnostic accuracy when evaluating rheumatologic cases [19]. Notably, using a version of an SC that incorporates diagnostic results, 33% of patients with rare diseases in the study of Ronicke et al [2] could have been correctly diagnosed on their first visit, significantly reducing the time to diagnosis.

These promising results align with the broader trend of increasing public acceptance and use of SCs. In the last decade, several SCs have been developed. In Germany alone, between 6.5% and 13% of adults have used an SC at least once [20,21]. A study involving over 1000 patients revealed that 63% of them would use a trusted SC, with a significantly higher willingness among those younger than 40 years of age compared to those older than 70 years [14].

While AI-powered SCs have shown promise in improving diagnostic accuracy and potentially patient outcomes, they face significant difficulties, particularly when it comes to rare diseases. The approach of SCs often involves extracting medical knowledge from vast amounts of data, often obtained through comprehensive literature reviews [16]. However, there is only limited research data and literature available on rare diseases, which makes them a particular challenge. This scarcity of data makes it difficult to source accurate information and, consequently, to model these diseases effectively within SCs. The variability in how rare diseases manifest in different patients adds another layer of difficulty. With symptoms that can vary significantly in severity, onset, and progression, modeling these diseases requires a more dynamic and flexible approach than is typically necessary for more common diseases.

Given these constraints, there is a need to explore and develop new methods to enhance the representation of rare diseases within SCs. Improving the diagnostic performance of SCs for rare diseases is not only crucial for individual patient outcomes but also for reducing the overall burden on health care systems by minimizing misdiagnoses and the associated unnecessary tests and treatments.

Lysosomal storage disorders (LSDs), a group of rare inherited metabolic disorders characterized by the accumulation of toxic substrates within the lysosomes [22], present an ideal case study for testing and refining these methodologies due to their complex and varied symptomatology. They require comprehensive treatment from a multidisciplinary team of neurologists, ophthalmologists, nephrologists, cardiologists, otorhinolaryngologists, pediatricians, geneticists, and dermatologists [23].

### Objectives of the Study

To address the challenges of modeling rare diseases in SCs, we conducted an exploratory pilot study with the following objectives: (1) to enhance the representation of Fabry disease within an SC by incorporating insights from guided interviews with experts. These insights were translated into clinical vignettes and used to optimize the disease model within the SC; and (2) to assess the performance of the newly optimized disease model by conducting symptom assessments and delivering questionnaires to patients with Fabry disease. This objective focused on determining whether the integration of guided interviews, in combination with literature review, results in improved diagnostic accuracy and patient satisfaction compared to models based solely on literature review.

## Methods

### Ethical Considerations

This mixed methods prospective pilot study was approved by the ethics committee of Hannover Medical School, Germany (10363_BO_K_2022), with patient enrollment between May 2022 and June 2023. The study was conducted in compliance with the Declaration of Helsinki and Good Clinical Practice. Written informed consent was obtained from all study participants with the possibility to opt out. No identifying participant information is presented in this study.

### Study Design and Setting

In the first phase, physicians were included as medical experts for the creation of the clinical vignettes when they met the criteria of having at least 10 years of clinical experience in the field of Fabry disease. In the second phase, this study included patients aged 18 years and older, experiencing Fabry disease, and fluent in German. Diagnosis of Fabry disease was defined as molecular genetic detection of any α-galactosidase A (GLA) gene mutation. Patients were randomized into 1 of 2 groups, with both patients and study physicians being blinded. Recruitment was conducted at the outpatient clinic of Nephrology and Pediatrics of Hannover Medical School. The SC Ada was selected for this study.

All interviews were recorded for quality assurance purposes. This study was conducted in collaboration with a German patient organization, Morbus Fabry Selbsthilfegruppe e.V., which provided valuable suggestions.

### Phase 1: Creation of Clinical Vignettes Through Expert Interviews

The guided interviews were conducted with 2 medical experts (JK and AD) specialized in Fabry disease from Hannover Medical School in Germany. The median time of professional experience in the field of LSDs was about 20 years for each expert.

The aim was to create clinical vignettes—structured case descriptions or scenarios—that typically include a patient’s medical history, symptoms, and relevant clinical details presented in a concise and standardized format. To ensure clinical realism and generalizability, experts constructed prototypical patient profiles based on their cumulative clinical experience rather than model vignettes on individual real cases. Demographic variables and symptom constellations were deliberately combined to reflect typical presentations of Fabry disease as seen in primary care, with the goal of capturing commonly observed patterns that could be recognized by SCs.

The interviews followed the structure of the clinical vignette template, beginning with the assessment of key demographics, including biological sex, age, pregnancy status (if applicable), smoking status, and history of high blood pressure, diabetes, or other known diseases. After establishing the demographic and medical history, the experts were asked to describe 1 or several primary complaints—symptoms that a patient would typically report when booking an appointment—as well as additional symptoms a patient might confirm or deny when directly questioned by a physician.

Experts were asked to select an appropriate urgency advice level for their presented case constellation. They could choose from an 8-level scale ranging from managing their symptoms at home to calling an ambulance.

The experts were then asked to assign potential differential diagnoses they would deem acceptable in view of the symptom constellation. Finally, they were asked whether any of the symptoms reported would present a very typical symptom, whose presence would lead the expert immediately to conclude that Fabry disease was the cause of the symptoms. The clinical vignette template, including all 8 possible urgency advice levels, can be found in Multimedia Appendix 1.

To ensure that the vignettes could be used effectively to optimize the Fabry disease model, the experts were instructed to mention only those symptoms that patients themselves could and would report. This is important because SCs generally rely solely on self-reported symptoms and do not take into account professional findings such as laboratory results or imaging techniques [11].

Interviews were conducted by the study physician (AP), a rheumatology resident employed by Charité Universitätsmedizin Berlin. The interviews were translated into English to enable integration of the information into the SC’s knowledge base. The translation was performed by a second study physician (NM-B), a German native-speaking employee of the evaluated SC developer who was familiar with the SC’s medical knowledge base to ensure that no information was lost during the translation process. Following the guided interviews, 5 clinical case vignettes were created. These vignettes were then integrated into the Ada SC’s medical knowledge base by converting them into structured, machine-readable information. The SC developer continuously monitors its SC performance by automatically running a large validation test case set and evaluating these against defined thresholds. Updating the Fabry disease model did not have any negative impact on the SC’s performance metrics.

The vignettes, along with insights from a structured literature review—a standard method for acquiring knowledge for SCs—were used to update the existing Fabry disease model. The processing of the Fabry disease model within the SC’s knowledge base was done independently by physicians employed by the SC software company. The study team was not involved in this process. A sample Fabry disease vignette is provided in Multimedia Appendix 2.

### Phase 2: Comparison of the Optimized Model With the Previous Fabry Disease Model

The objective of this phase was to compare the preoptimized SC version, which contained the original Fabry disease model based on a literature review, with the optimized version. The comparison focused on performance metrics, including diagnostic accuracy and overall user satisfaction. The disease model comparison was facilitated by 1 of the 2 study physicians (AP and NM-B), with one of them always being on site. Two tablets were prepared with a study version of the SC—one containing the original Fabry disease model and the other featuring the newly optimized model. The study version of the SC was identical to the on-market version in German, with the only difference being that the study team could select earlier versions of the medical knowledge and medical models, a prerequisite for this direct comparison. The specific tablet, and thus the version of the SC used first, was randomly assigned by the study physicians. The study was conducted in a double-blind manner, ensuring that neither the 2 study physicians nor the participants knew which tablet had which version of the SC. Blinding of study physicians was maintained until the conclusion of the data analysis. To begin the symptom assessment, study participants were asked to enter the symptoms that had most troubled them at the onset of their illness. They were then guided through an AI-generated sequence of questions, where they were instructed to confirm the symptoms they had experienced during their patient journey and to deny those they had not. An “I don’t know” option was also available for any uncertainties. Upon completion, participants received a symptom report summarizing their responses, along with a list of potential diseases that could be causing their symptoms, including their probabilities and recommended next steps. The report additionally provided them with further information about the possible diseases.

After completing the first assessment, participants performed another assessment using the second tablet with the other disease model. Study physicians did not interfere with the symptom assessment in any way after receiving the consent for study participation, in order not to influence the assessment.

### Questionnaires

Following each symptom assessment, participants were asked to complete a questionnaire to evaluate the quality of the assessment, whether their illness was listed as one of the proposed disease suggestions by the SC, and whether the questions were easy to understand. The second questionnaire contained an additional question asking which assessment the participants preferred. The English version of the first questionnaire is provided in Multimedia Appendix 3.

### Data Analysis

The primary objective of the second part of this study was to compare the diagnostic accuracy of 2 SC versions: one with the Fabry disease model developed on the basis of a literature review, while the other was additionally trained with case vignettes from experts. Diagnostic accuracy was evaluated using Matching scores, commonly referred to as *M* scores. *M* scores represent the degree to which the model’s diagnostic output aligns with the correct diagnosis, serving as a metric to assess the model’s accuracy in identifying the correct diseases, also referred to as conditions in the context of SCs [11]. All suggested differential diagnoses generated by the model are ranked according to their likelihood. The *M*1 score specifically measures the accuracy of the model’s top-ranked disease. In other words, *M*1 indicates how often the first disease proposed by the model is the correct diagnosis. The *M*3 score assesses the model’s accuracy by determining whether the correct diagnosis is among the top 3 suggested diseases, offering a broader evaluation of the model’s diagnostic performance.

With the help of descriptive statistics, we compared overall satisfaction and perceived completeness of symptom coverage between the original and optimized versions of the SC. Participants were asked which version they preferred, and satisfaction scores were determined using a 4-point Likert scale. The scores were analyzed both collectively and individually for each patient to identify which version was rated higher.

We evaluated how completely each version of the SC covered the patients’ symptoms and how helpful participants thought the SC would have been if it had been used at the onset of their illness. Responses were categorized by the degree of completeness and helpfulness for each version. Additionally, we performed a Wilcoxon signed rank test to compare satisfaction scores between the original and optimized versions of the SC.

## Results

### Comparison of the Optimized and Original Fabry Disease Model

**Figure 1.**
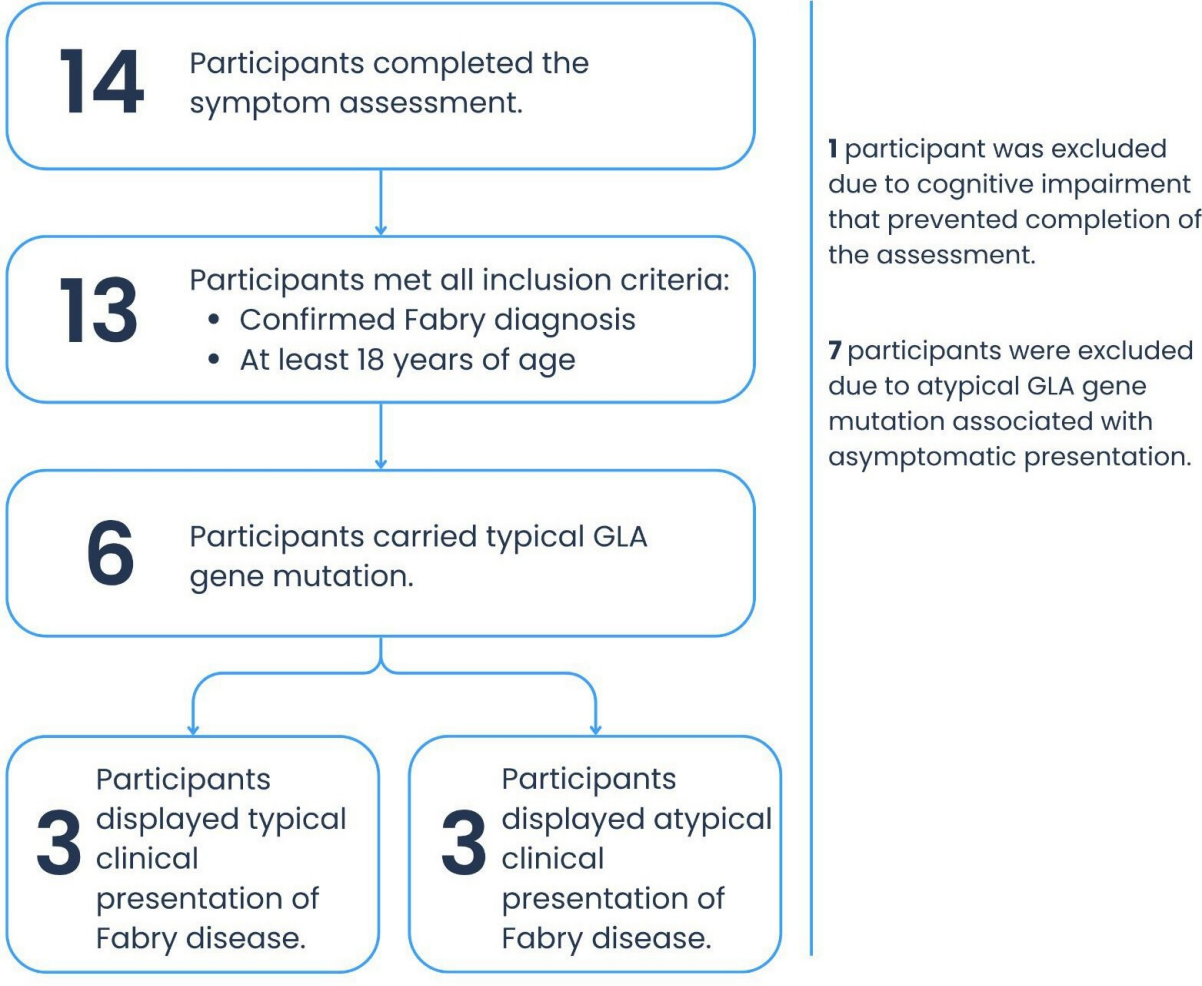
Participant recruitment flowchart. GLA: α-galactosidase A.

Between May 2022 and June 2023, 14 patients with Fabry disease were enrolled to compare the diagnostic accuracy and user satisfaction between the optimized and original SC versions. In total, 12 of the 14 patients were female, and 2 were male. A total of 7 patients were excluded from the final analysis as they had atypical GLA gene mutations and therefore were either asymptomatic or had very atypical symptoms. One patient had to be excluded because of a diagnosed cognitive deficit that affected his ability to complete the study. This left 6 patients with typical mutations for the final analysis, 3 of whom reported typical Fabry-related symptoms, while the other 3 reported atypical symptoms. Figure 1 shows the participant recruitment flow.

### Comparison of Diagnostic Accuracies for the Original and Optimized Versions

Regarding the top disease accuracy (*M*1), the original SC version identified Fabry disease as the top suggestion in only 1 of 6 (17%) cases. The optimized version improved this, identifying Fabry disease as the top disease in 2 of 6 (33%) cases.

In 3 of 6 participants, both SC versions listed Fabry disease among the first 3 disease suggestions, yielding an *M*3 score of 50%. In all 3 patients where the SC suggested Fabry disease, characteristic symptoms such as acroparesthesia, angiokeratoma, or hypohidrosis were present.

### Comparison of Participant Satisfaction Between the Original and the Optimized Versions

When asked which SC version they preferred, 3 patients chose the optimized version, 2 the old one, and 1 patient was indecisive. Overall, the optimized Fabry disease model received higher total ratings (108 vs 103). Individually, 3 patients rated the optimized version highest (12 vs 9; 17 vs 13; and 23 vs 20), 2 patients gave equal scores to both versions (21 vs 21 and 21 vs 21), and 1 patient rated the original version highest (14 vs 19). This comparison is displayed in Table 1. The Wilcoxon signed-rank test indicated no statistically significant difference in satisfaction ratings between the optimized and original SC versions (*W*=4.0; *P*=.71).

**Table 1.** Overall score ratings per patient per symptom checker version.

	Original version	Optimized version
Participant 1	9	12
Participant 2	19	14
Participant 3	13	17
Participant 4	21	21
Participant 5	20	23
Participant 6	21	21

Regarding symptom coverage, the optimized version was rated more favorably in terms of completeness, with patients describing it as “complete” 3 times, “almost complete” twice, and “somewhat complete” once. In contrast, the original version’s symptom coverage was described once as “complete,” once as “almost complete,” thrice as “partially complete,” and once as “somewhat complete.”

When asked how helpful the SC would have been at the onset of their disease, all 3 participants for whom Fabry disease was listed as a possible cause of their symptoms rated both versions as “very helpful.” The 3 patients where Fabry disease was not listed considered the SC either “partially helpful” (2/3) or “somewhat helpful” (1/3).

## Discussion

### Summary of the Findings

The optimized SC version, enhanced with expert knowledge, demonstrated improved diagnostic accuracy for Fabry disease compared to the original version. The optimized version identified Fabry disease as the top disease in 33% (2/6) of the cases (*M*1 score), compared to 17% (1/6) in the original version. The *M*3 scores were consistent across both the original and the optimized versions, with Fabry disease being listed among the top 3 suggested diseases in 50% (3/6) of the cases.

Patients generally preferred the optimized SC version, rating it higher in view of completeness and overall satisfaction. The optimized version was often described as providing more comprehensive symptom coverage and was perceived as more helpful if it had been available at the onset of their disease.

### Improving Diagnostic Accuracy

The diagnostic accuracy of SCs is crucial in the context of rare diseases, where timely and accurate diagnosis remains a significant challenge, often leading to a lower quality of life and reduced life expectancy [24]. In this study, the optimized SC model showed a modest improvement in identifying Fabry disease as the top diagnostic suggestion, with the *M*1 score increasing from 17% (1/6) to 33% (2/6). While this improvement may seem minor, even slight enhancements in diagnostic accuracy can have a substantial impact on patients with rare diseases. Given the prolonged diagnostic odyssey often associated with these diseases, any increase in accuracy can facilitate earlier intervention, which is vital to prevent disease progression and improve long-term outcomes.

The study also highlights the main limitations of SCs, in particular their dependence on clear and well-defined symptoms. SCs rely heavily on user-provided data, typically self-reported symptoms, and are therefore most effective when patients present with symptoms that align closely with the embedded diagnostic algorithms. This study demonstrated that SCs struggle to accurately identify patients with few or nonspecific symptoms, a common scenario in the early stages of many rare diseases. For example, patients with Fabry disease who were primarily diagnosed on the basis of family history, rather than clear symptom profiles, were not effectively identified by the SC.

Our findings are consistent with the broader challenges of diagnosing atypical or uncommon diseases—precisely where accurate identification is most needed [25]. The SC in this study, like others, had lower diagnostic accuracy for atypical presentations, emphasizing the need for continuous refinement to better recognize these cases. Expanding the range of clinical vignettes, especially those depicting atypical scenarios, could help address this gap. In this study, experts primarily focused on creating vignettes of typical cases, which inadvertently led to the omission of more atypical presentations. Incorporating a diverse array of both typical and atypical cases is crucial for broader diagnostic coverage, though developing and integrating such vignettes presents a challenge for SC software companies.

While SCs may currently struggle to identify patients with minimal or nonspecific symptoms, they play a pivotal role in prescreening and encouraging consideration of less obvious diagnoses for those with oligosymptoms. By providing an initial assessment based on reported symptoms, SCs can help identify potential rare diseases early in the diagnostic process. This prescreening function empowers patients by giving them a clearer understanding of their symptoms and facilitating timely and appropriate care-seeking behavior.

Moreover, SCs are increasingly valuable in supporting health care providers, particularly GPs, who may lack the specialized knowledge needed to accurately diagnose rare diseases [26,27]. By highlighting potential rare diseases, SCs can prompt clinicians to consider diagnoses that may not be immediately apparent, thereby improving the overall diagnostic process. This guidance is especially valuable for ensuring that patients are promptly referred to specialists or undergo further diagnostic testing, thereby reducing the time to an accurate diagnosis. Assessing the combined diagnostic accuracy of SCs and physician expertise is a promising approach that better reflects the reality of clinical care, where the diagnostic process goes beyond the initial use of SCs by patients. This integrated approach recognizes the critical role of clinicians in interpreting the results of SCs to make informed decisions that ultimately lead to more effective patient care.

Improving the modeling of rare diseases within SCs is one approach to enhancing diagnostic accuracy. Another strategy involves integrating more comprehensive information into the SC, for example, by incorporating large language models (LLMs) that can facilitate more accurate information intake from patients, including interpreting patient-provided notes or accessing electronic health records. LLMs can analyze and synthesize patient data more effectively, providing a richer context for the SC to generate accurate differential diagnoses.

Additionally, SCs could gradually incorporate more detailed patient information, such as laboratory results and other diagnostic data, into their analyses. This would enable the refinement of algorithms to deliver more accurate and contextually relevant diagnoses. The collaboration between LLMs and SCs could create a powerful diagnostic tool, where LLMs enhance the understanding of complex patient inputs, and SCs apply this information to produce more reliable and timely diagnoses. This combined approach could significantly improve the accuracy and effectiveness of SCs, particularly for diagnosing rare and complex diseases.

### User Satisfaction

In the blind comparison of both models, users preferred the optimized version, regardless of which was shown first. This preference is probably due to the fact that the optimized version can provide more accurate diagnostic suggestions, which resonated more with users. Users tended to prefer the optimized version, reporting that the optimized version asked more relevant questions and better covered their symptoms, even when the SC did not identify Fabry disease as the likely cause of their symptoms. Those users experienced atypical symptoms. This shows that optimizing the Fabry condition model, together with improving the wording of associated symptoms, using the clinical vignettes, has increased the SC’s ability to understand user input, which may have contributed to overall user satisfaction. The streamlined questioning process may also have played a role. As users interact with the app, each response updates the app’s internal differential diagnoses, which in turn refines the subsequent question flow. If the optimized version identifies the correct disease earlier in the process, it can streamline the experience by reducing unnecessary questions and focusing more quickly on relevant diagnostic paths. This could result in a more efficient and satisfying interaction.

### Strengths and Limitations

#### Strengths

Our study has several strengths. One of the primary ones is the double-blind approach used for study phase 2. By ensuring that neither the participants nor the study physicians knew which version of the SC they were using, we minimized bias and ensured that the observed preferences for the optimized model were based purely on its performance and not on any preconceived notions.

The study focuses on Fabry disease, a complex and rare disease, which makes it even more relevant. Fabry disease poses significant diagnostic challenges and is therefore an ideal subject for evaluating the effectiveness of SCs, particularly in the context of rare diseases where early and accurate diagnosis is crucial.

Another innovative aspect of our study is the use of expert-derived clinical vignettes as a data source for enhancing the SC’s diagnostic algorithms. Unlike the traditional use of vignettes, which typically serve as evaluation tools, we used them to directly improve the underlying algorithms.

Working with the German patient organization Morbus Fabry Selbsthilfegruppe eV provided further invaluable insights that ensured that the study remained closely aligned with the needs and concerns of patients. This partnership helped to guide the study’s focus and ensured that the results were meaningful and beneficial to both patients and health care providers.

#### Limitations

Our study has several limitations that should be acknowledged. One of the primary limitations is the gender imbalance in the patient population. Due to the limited availability of patients from the outpatient clinic, the study predominantly included female patients with Fabry disease (12 of 14 participants). Fabry disease is inherited in an X-linked pattern, meaning that female patients often present with less severe symptoms or may even be asymptomatic. This gender imbalance affected the results of the study since the SC relies heavily on symptoms reported by patients. This limitation highlights the need for future studies to include a more balanced patient population, particularly with more male patients who typically show more pronounced symptoms. The results may have been affected by the fact that the patients with Fabry disease who performed the SC version comparison may not have been able to remember the symptoms they had experienced at an earlier stage. They may have only entered their current symptoms, whereas Fabry disease symptoms become more severe over time [26]. At the same time, most patients interviewed were receiving enzyme replacement therapy at the time of study participation. It is possible that these facts affected the SC’s diagnostic accuracy for Fabry disease; however, it is difficult to determine to what extent.

We will address these limitations in future studies by including GPs experienced with LSDs and patients who are at an earlier stage in their diagnostic journey. Such studies will allow us to assess the impact of SCs and our new approach of enriching SC disease models with expert knowledge for the detection of LSD in primary care.

Another limitation of the study is the small sample size. Of the 14 initially enrolled patients, 8 (57%) had to be excluded from the final analysis due to atypical GLA gene mutations, which led to very atypical or even asymptomatic presentations of Fabry disease. An essential prerequisite for the effective use of an SC is that the patient has at least some symptoms. Many of the excluded patients were diagnosed based on a positive family history rather than symptomatic presentation, which is common in Fabry disease where a male family member with typical symptoms is often the index patient. The small sample size limits the generalizability of the study’s findings and suggests the need for refining inclusion criteria in future studies to better reflect the real-life presentation of Fabry disease.

To test the accuracy of an SC, previous studies often imitated real patient input in the form of clinical vignettes used by either patients or health care professionals to enter SC symptoms [11,17,18,28,29].

Although these studies use vignettes for the assessment of SCs, which is different from the use of vignettes in our study, we have nonetheless informed our own study design by acknowledging the limitations reported in these studies. Vignettes have some limitations when used as clinical evaluation approaches, as they have limited information content and do not generally provide the opportunity to clinically interrogate additional information, to examine the patient, or to assess nonverbal cues [29,30]; therefore, at the time of vignette input into the SC, assumptions must sometimes be made beyond the vignette script. In addition, vignettes do not perfectly reflect how real patients use SCs [27]. Despite these limitations, clinical vignette studies are widely applied in the evaluation of SCs, as the approach also offers advantages since it allows 1 or more SCs to be evaluated (comparatively) across a broad spectrum of clinical conditions and clinical presentations using a highly standardized study protocol. The limitations of vignettes can be minimized through careful vignette standardization and through careful design, review, and refinement [11]. The use of vignettes is particularly important in the field of rare diseases, as these conditions are uncommon and cannot be readily addressed using conventional clinical studies without very large participant numbers or the preselection of participants highly likely to have the conditions under investigation.

In contrast to the standard use of vignettes for SC assessment, our study recruited real patients with Fabry disease and asked them to enter their symptoms into the app. We took substantial care in the development of our vignettes to follow best practices and to develop vignettes that were comprehensive in their description of the clinical presentation. Our goal was to provide repeatable and accurate input, even though the primary purpose was to use the vignettes as additional evidence for disease model optimization as opposed to SC evaluation. Although using vignettes for this purpose is a well-established method [11,18] and vignettes can be of high quality due to their elaborate creation process [11], they may not perfectly reflect how real patients use SCs [31].

Additionally, one of the study physicians was employed by the SC software company at the time of the study. Although this physician did not interfere with patients with Fabry disease entering their symptoms during the SC version comparison, this affiliation may introduce potential bias. To mitigate this, future evaluations should aim to include independent studies with larger numbers of participants to validate the findings more robustly.

### Conclusions

This study demonstrates that integrating expert-derived clinical vignettes into AI-powered SCs can improve diagnostic accuracy and user satisfaction, particularly for rare diseases such as Fabry disease. The optimized SC version, which incorporated these vignettes, showed improved performance in identifying Fabry disease as a top diagnostic suggestion and received higher user satisfaction ratings compared to the original version. However, to fully realize the potential of this approach, it is crucial to include vignettes representing atypical presentations to ensure broader diagnostic coverage. Additionally, larger-scale studies are necessary to validate these findings, given the small sample size in this pilot study. Expanding the scope of this method could offer a more robust tool for early diagnosis and patient care in rare diseases.

## Supplementary material

10.2196/55001 Multimedia Appendix 1 Clinical vignette template.

10.2196/55001 Multimedia Appendix 2 Clinical vignette Fabry.

10.2196/55001 Multimedia Appendix 3 Questionnaire.

## Abbreviations

AI: artificial intelligence
GLA: α-galactosidase A
GP: general practitioner
LLM: large language model
LSD: lysosomal storage disorder
SC: symptom checker
