# Supplementary material for: Medical Expert Knowledge Meets AI to Enhance Symptom Checker Performance for Rare Disease Identification in Fabry Disease: Mixed Methods Study
Source: JMIR AI. 2025 Aug 28;4:e55001. doi: 10.2196/55001 (PMC12392689; doi:10.2196/55001)
Supplement: Multimedia Appendix 2 [file ai-v4-e55001-s002.pdf]

<u>Clinical Vignette Fabry</u>

Please bold or highlight answer selection and type freeform answers. Initially we are looking for classical or typical presentations, but it can also be helpful to have rare or challenging cases.

**Gender**	Male / Female
**Age**	28 years
**Previous medical history** (key information and risk factors)	
Are they pregnant?	Yes / No / I'm not sure
Are they a smoker?	Yes / No / I'd rather not say
Have they ever been diagnosed with high blood pressure?	Yes / No / I'm not sure
Do they have diabetes?	Yes / No / I'm not sure
Have they been diagnosed with/treated for any other illness? This could include past surgeries, conditions, or treatments.	Yes / No / I'm not sure
**Primary complaint**<br><br>(Symptom most troubling to the patient, ideally in lay terminology. If it is a collection of symptoms then put these here and then further related histories to them below)	Mostly pain in hands and feet<br>→ Change of pain in course of disease<br><br>Angiokeratoma
**Additional information from patient**<br><br>*(That's what the patient says on his own when he's at the doctor)*	Since 5 years pain in wrists and ankles<br><br>Angiokeratoma in belly button
**Additional information on primary complaint and current symptoms**<br><br>(E.g., how long has primary complaint troubled the patient? Are there other symptoms troubling the patient? You can provide a detailed or narrative-like	Hypohidrosis<br><br>Gastrointestinal complaints (diarrhea + abdominal<br><br><br>Professional:<br>Tortuositas vasorum

account here. It should cover positively associated symptoms that rule “in” the diagnosis and negatively associated ones, the help rule it “out”.)	
**Advice level**<br><br>(What would your recommended care advice be for this particular presentation?)	Self-care /<br>Self-care (pharmacy) /<br>Primary care (within 2-3 weeks) /<br>Primary care (within 2-3 days) /<br>Primary care (same day) /<br>Primary care (within 4 hours) /<br>Emergency care /<br>Call ambulance
**Main diagnosis**<br><br>(What was the final diagnosis for this case? Please provide the ICD-10-CM code if possible.)	Fabry disease
**Differential diagnoses**<br><br>(List at least three to four other conditions that could cause these symptoms, from highest to lowest probability with ICD-10 codes)	Rheumatoid Arthritis<br>Neurological symptoms (small fiber e.g.)
**Red Flags**	Burning pain<br>Angiokeratoma<br>Hyperhidrosis<br>Proteinuria<br>Right heart failure
