# Supplementary material for: Medical Expert Knowledge Meets AI to Enhance Symptom Checker Performance for Rare Disease Identification in Fabry Disease: Mixed Methods Study
Source: JMIR AI. 2025 Aug 28;4:e55001. doi: 10.2196/55001 (PMC12392689; doi:10.2196/55001)
Supplement: Multimedia Appendix 3 [file ai-v4-e55001-s003.pdf]

Questionnaire #1: For Fabry patients

Who: Patients diagnosed with the lysosomal storage disease (LSD) *Fabry disease*

When: During a visit to the MHH Specialty Center, the patient will complete the questionnaire immediately after finishing the Ada symptom assessment

Goal: This questionnaire will serve as an assessment of Ada's medical models. Your participation will serve the larger goal of improving diagnosis for others with similar conditions in the future.

Please note: The Ada symptom assessment report aims to inform your doctor of your complaints, the questions Ada asked you, Ada's suggestions that may cause these symptoms, and educational information provided with the report results. The assessment is **not intended to drive diagnostic decision-making** or to override the treating physician's planned diagnostic workup. *Please keep Ada's Intended Purpose in mind when assessing 'appropriateness' below.*						
**1.** Which lysosomal storage disease (LSD) were you previously diagnosed with by a healthcare professional?	**Fabry (0)**					
**2.** Was your final diagnosis (Fabry, Gaucher, Pompe) an ***exact match*** with one condition in Ada's list?	**No (0)**	**Yes (1)**				
**2a.** Which position was the final diagnosis in the list?	**First (1)**	**Second (2)**	**Third (3)**	**Fourth (4)**	**Fifth (5)**	
**2b.** How useful was the information given in the report about your final diagnosis?	**Not at all useful (0)**	**Somewhat useful (1)**	**Moderately useful (2)**	**Useful (3)**	**Very useful (4)**	**N/A (5)**

**2c.** Did Ada provide a condition suggestion matching *a condition you know to be* ***closely related*** to the diagnosis you received from your doctor (e.g. another Lysosomal Storage Disease)?	**No (0)**	**Yes (1)**				
**3.** Overall, how *satisfied* are you with the Ada symptom assessment?	**Not at all satisfied (0)**	**Somewhat satisfied (1)**	**Moderately satisfied (2)**	**Satisfied (3)**	**Very satisfied (4)**	
**4.** Considering your final diagnosis, how *appropriate* do you think Ada's list of suggested conditions is?	**Not at all appropriate (0)**	**Somewhat appropriate (1)**	**Moderately appropriate (2)**	**Appropriate (3)**	**Very appropriate (4)**	
**5.** How well do you think Ada collected the symptoms related to your condition?	**Not at all satisfied (0)**	**Somewhat satisfied (1)**	**Moderately satisfied (2)**	**Satisfied (3)**	**Very satisfied (4)**	
**6.** How well did the questions capture your range of symptoms?	**Not at all complete (0)**	**Somewhat complete (1)**	**Moderately complete (2)**	**Complete (3)**	**Very complete (4)**	
**7.** Imagine you had completed this symptom assessment earlier in your diagnostic odyssey: how *helpful* do you imagine Ada would have been?	**Not at all helpful (0)**	**Somewhat helpful (1)**	**Moderately helpful (2)**	**Helpful (3)**	**Very helpful (4)**	

**8.** For the entire list of condition suggestions, how useful was the information given in the report?	**Not at all useful (0)**	**Somewhat useful (1)**	**Moderately useful (2)**	**Useful (3)**	**Very useful (4)**	**N/A (5)**

**9.** Do you have any additional comments or feedback about the Ada assessment report?	

Thank you for your participation!
